# Negative Effects of “Predatory” Journals on Global Health Research

**DOI:** 10.29024/aogh.2389

**Published:** 2018-11-05

**Authors:** Diego A. Forero, Marilyn H. Oermann, Andrea Manca, Franca Deriu, Hugo Mendieta-Zerón, Mehdi Dadkhah, Roshan Bhad, Smita N. Deshpande, Wei Wang, Myriam Patricia Cifuentes

**Affiliations:** 1 Laboratory of NeuroPsychiatric Genetics, Biomedical Sciences Research Group, School of Medicine, Universidad Antonio Nariño, Bogotá, CO; 2 PhD Program in Health Sciences, School of Medicine, Universidad Antonio Nariño, Bogotá, CO; 3 Thelma M. Ingles Professor of Nursing, Duke University School of Nursing, Durham, North Carolina, US; 4 Department of Biomedical Sciences, University of Sassari, Sassari, IT; 5 Faculty of Medicine, Autonomous University of the State of Mexico (UAEMex), Toluca, MX; 6 Department of Management, Faculty of Economics and Administrative Sciences, Ferdowsi University of Mashhad, Mashhad, IR; 7 All India Institute of Medical Sciences (AIIMS), New Delhi, IN; 8 Department of Psychiatry, De-addiction Services and Resource Center for Tobacco Control, Centre of Excellence in Mental Health PGIMER-Dr. Ram Manohar Lohia Hospital, New Delhi, IN; 9 School of Medical Sciences, Edith Cowan University, Joondalup, AU; 10 Beijing Municipal Key Laboratory of Clinical Epidemiology, Capital Medical University, Beijing, CN; 11 Department of Mathematics, School of Sciences, Universidad Antonio Nariño, Bogotá, CO; 12 Laboratory of NeuroPsychiatric Genetics, School of Medicine, Universidad Antonio Nariño, Bogotá, CO

## Abstract

Predatory journals (PJ) exploit the open-access model promising high acceptance rate and fast track publishing without proper peer review. At minimum, PJ are eroding the credibility of the scientific literature in the health sciences as they actually boost the propagation of errors. In this article, we identify issues with PJ and provide several responses, from international and interdisciplinary perspectives in health sciences. Authors, particularly researchers with limited previous experience with international publications, need to be careful when considering potential journals for submission, due to the current existence of large numbers of PJ. Universities around the world, particularly in developing countries, might develop strategies to discourage their researchers from submitting manuscripts to PJ or serving as members of their editorial committees.

## Background on predatory journals

Research quality faces multiple threats, from improperly designed studies, ethical concerns, biased results, and growing publication costs to unfair judgment prior to scientific publishing. As strong evidence from research results might inform decision making, independently or as a chain with domino effects, in the field of health, all threats can affect the well-being of individuals and populations. The industry of research publishing is evolving according to broad available electronic means and increasing amounts of research to handle. A growing trend of open access (OA) publishing that shifts publication costs to authors has opened the door to money as a mediator that could even surpass quality assessment by peer review.

The term “Predatory Journals” (PJ) first appeared in PubMed in 2012, from a note published in the *Nature* journal by Professor Jeffrey Beall [1]. For years, Professor Beall maintained and updated online list of potential, possible, or probable predatory publishers and journals, and he had proposed several criteria for their identification, such as the use of massive email spam asking for article submissions or for joining the editorial board, absence or misrepresentation of the publisher’s headquarters’ location, and lack of copyediting in the published articles, among others [2]. According to the last available version of Beall’s list (January 2017), 1,155 publishers (from a number of 18 in 2010) and 1,294 standalone journals were included. In this article, we revisit the phenomenon of predatory publishing, gathering a general landscape of it for science in general and scanning its effects on global health. We provide an update according to recent challenges worldwide and that global health researchers face to protect themselves from predatory publishing and the growth of the problem.

Predatory publishers have several strategies to “invite” scholars to publish in their journals that should alert researchers to this type of publication. One of the most common practices is to send and re-send hundreds or thousands of e-mails around the world, expecting to find some potential authors. Newer scholars from developing countries are particularly at risk of becoming the victims of these practices [3]. As publication costs of recognized OA journals are not affordable for a large number of researchers in developing countries, lower fees can encourage authors to send a paper to a journal of this kind. In this context, the expensive fees charged on average by mainstream OA journals may have played a role in the proliferation of PJ, which deceptively pose themselves as low-cost alternatives.

Predatory journals are publications that exploit the OA model by asking authors to pay directly or indirectly for publication in the journal. These journals promise high acceptance rates and fast-track publishing and often report unauthentic impact factors. Predatory journals often publish papers in few days or weeks, much quicker than a standard peer review process, and typically have no editorial office or well-recognized institution/organization associated with the journal. Consequently the manuscripts have significant grammatical and typographical errors and the layout often looks unprofessional [4]. Names of predatory journals are usually quite broad, for example *Journal of Sciences* or *International Journal of Science*, aiming to attract more submissions. Often only the first or early issues are available [2,5]. Sometimes the publisher’s website advertises sub-standard conferences and, accordingly, result in predatory conference proceedings.

Recently some researchers have proposed a predatory ranking metric called “Predatory Rate” (PR), which is based on 14 criteria such as editorial members, review process and publishing, announcements, open access policy, and publication charges. Each criterion has a weight ranging from 1 to 3, and PR is a continuous value between 0 and 1. APR equal to 0 means that the journal is possibly not a predatory one. A PR higher than 0 and lower than 0.22 suggest that the journal uses predatory practices. The remaining values indicate the journal is possibly a predatory one [6]. However, this predatory metric rate omits some criteria for predatory journals, and more robust indices would make an outstanding contribution to screen predatory publishing [7]. It is important to highlight that universities and academic societies from developing counties offer their journals at low or no cost of publication [4]. Furthermore, the process required for a new journal to be included in various recognized indexing services, such as Medline and the Journal Citation Reports, is often complex and lengthy, sometimes requiring several years before being included. Thus, lack of inclusion of a journal in these search engines does not provide evidence that the journal is illegitimate, since it may be too new to be included [8]. In Table 1, we suggest some criteria (non-absolute and non-exhaustive) that illustrate some characteristics between mainstream and predatory journals.

**Table 1:** Several differences between mainstream and predatory open access journals.

Characteristic	Mainstream	Predatory

Peer review	Strict	Uncertain or absent
Costs to publish	High	Low
Location	Mainly in developed countries	Mainly in developing countries
Indexing	Recognized and with high qualifications	With less strict criteria or without indexing
Impact factor	High in subscription based journals	Low or absent
Editorial team	Recognized for their trajectory and position	Less known
Financial target	Lucrative or with high costs to be open access	Lucrative

Shen et al. estimated that PJ published more than 420,000 articles in 2014, from around 8,000 active journals [9], and Gutierrez et al. identified more than 20 spurious alternative impact factors, which were claiming to provide false metrics for PJ [10]. Dadkhah and colleagues inspected about 300 PJ to identify misleading metrics and found that they were associated with 34 different metrics [11] and that India, USA, Iran, Indonesia, and Turkey were the top five targeted countries [11]. In the latest available version of Beall’s list, 53 misleading metrics were included at the beginning of 2017. According to several PJ websites, the fact that a large number of members of their editorial boards are scientists working in North America is noticeable.

## Negative implications of predatory journals for global health sciences

The existence of PJ leads to a distortion of the published scientific literature, allowing the online existence of manuscripts that did not pass a rigorous process of peer review. In a study of PJ in nursing, as an example, the researchers found that many journal websites indicated that manuscripts were peer reviewed; however, the quality of those reviews was questionable, with some reviews done within one to two days and followed by rapid publication of the article without any editorial review or copyediting [12]. The negative impact of very-low quality publications is higher for health sciences around the globe, due to the possible direct implications on health care and research [13,14]. Without an adequate peer review process and limited editorial oversight, there are no mechanisms to verify if the quality of the articles is correct and avoid findings that can be potentially harmful to patients and others. At minimum, PJ are undermining the credibility of the scientific literature in the health sciences as they can promote the propagation of errors. Researchers might cite papers that have been published in PJ and discuss invalid findings in their articles submitted to reputable journals. Since PJ are often available free online, they have an unknown but surely detrimental effect on medical education as well as patient knowledge (since patients also roam the internet in search of information about their illnesses) [15].

Manca et al. identified 192 potential PJ in the fields of neurology and neurosciences, in addition to 59 potential PJ in the field of rehabilitation, with some of them (20% in neurology, 11% in neurosciences, 12% in rehabilitation) indexed in PubMed [16,17]. In a sample of 613 journals, Shen et al. identified that 27.1% of the publishers were from India and that 34.7, 25.6, and 16.4% of the corresponding authors were from India, Asia (without India), and Africa, respectively. In this study, the authors also identified that 9.2, 8.8, and 2.2% of the corresponding authors were from North America, Europe, and South America, respectively [9]. In an interesting experiment, Bohannon submitted a deeply flawed manuscript to 304 OA journals (including 161 journals from the Directory of Open Access Journals [DOAJ], 121 from Beall’s list and 16 from both) and found that it was accepted by 157 of them. He reported that the manuscript was accepted with no or superficial peer review by 84 journals included in Beall’s list and by 66 journals indexed in DOAJ [18]. Xia et al. analyzed seven journals from Beall’s list and found that an important fraction of corresponding authors had no previous publications and citations, with authors mainly from South Asia and Africa [19]. Sorokowski et al. sent applications, for a fictitious character, to be a member of editorial boards, and 33 and 7% of journals included in the list of PJ and DOAJ accepted it, respectively [20]. The requirement by some Medical Councils for a specific number of publications before being considered for promotion has benefited such predatory publishing. Regrettably, some publications with a lower evidence base (e.g. case reports) are in many cases merely to see authors’ own names in print, or for building up their list of publications. The mantra of ‘publish or perish’, the desire for exhibiting one’s name in print, or even the poor quality of teaching for medical writing, may also drive authors towards PJ [21]. To counteract such negative patterns, some national funding bodies and universities have incorporated the list of PJ into their black list of journals and publishers.

These journals also have negative effects on science integrity. They lead to spreading junk science [22] and can decrease academic ranking of countries. It is possible that when researchers from specific countries publish their works in PJ, the relative proportion of published papers for the mentioned countries will decrease in reputable indexed journals. This might lead to a lower position of countries in academic ranking systems, such as Scimago (http://www.scimagojr.com/).

## Recent events on list of predatory journals

**Figure 1:**
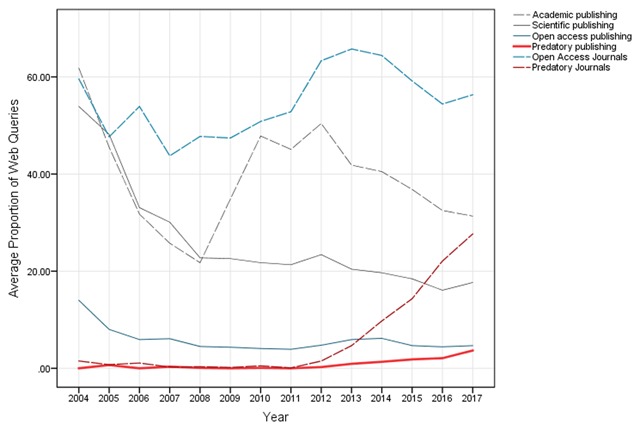
Google Trends of yearly web queries as proxies of general awareness about predatory publishing (PP) and predatory journals (PJ). Compared to trends of queries about predatory publishing and journals (red continuous and dotted lines), trends of web queries about open access publishing (OAP) and journals (OAJ) have more steady trends (blue continuous and dotted lines), are weakly correlated and significantly different (r_PP-OAP_ = 0.3; r_PJ-OAJ_ = –0.2; Both PP-OAP and PJ-OAJ comparisons had t-test p-values < 0.05). Trends of Academic and Scientific publishing were included as a reference (gray continuous and dotted lines).

**Figure 2:**
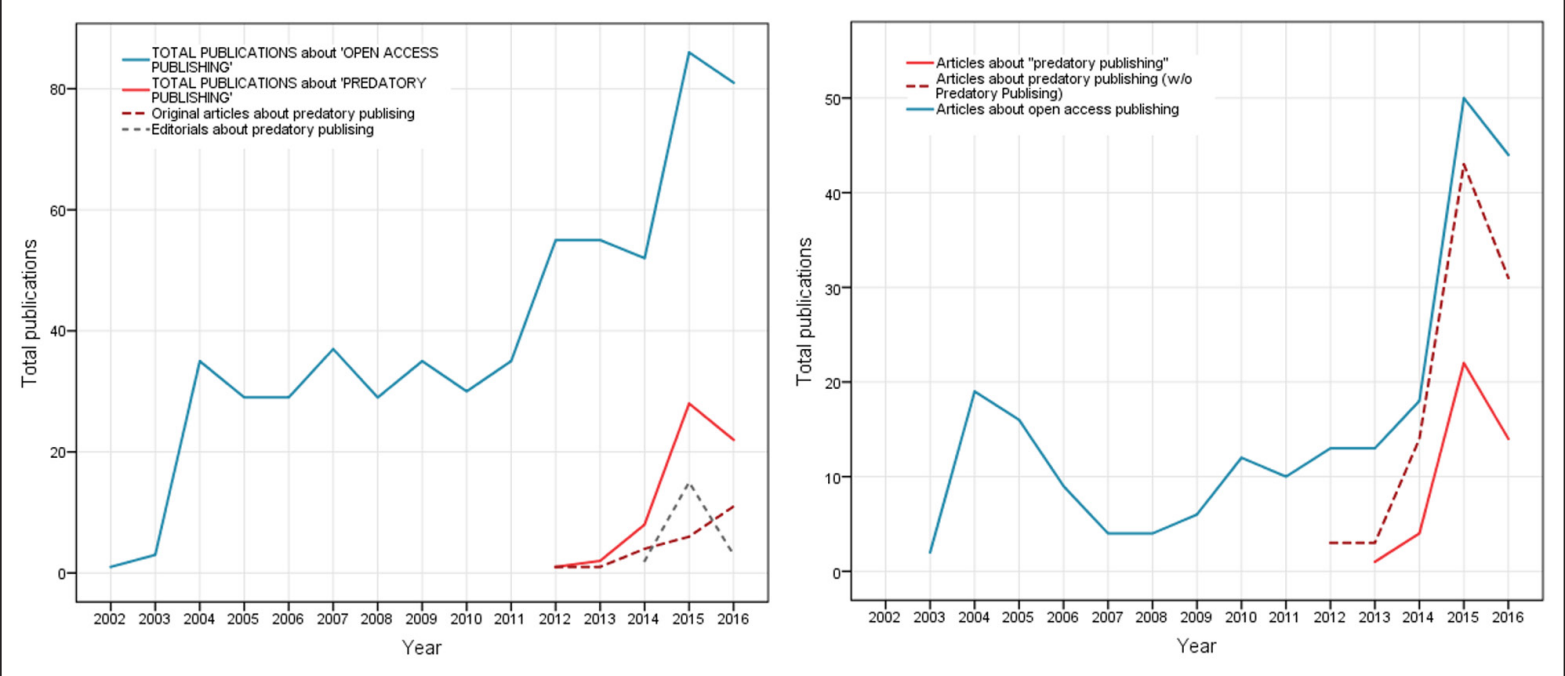
Scopus (Left) and PubMed (right) trend reports of number of articles about Predatory Publishing (PP, red) and Open Access (OA, blue) by year. Correlations between PP-OA trends were high (Scopus r = 0.72 PubMed r = 0.87). By t-tests, differences in Scopus were in the limit (p = 0.05) and in PubMed were non-significant (p > 0.05). In Scopus, the growing trend predominantly relies on original articles (dotted red line).

In January 2017, Beall’s list of predatory publishers and journals was no longer available [23]. Fortunately, the list are still available thanks to the Internet Archive (https://web.archive.org/web/20170103170903/https://scholarlyoa.com). Currently, the reasons for the disappearance of the Beall’s list are unknown. However, the United States Federal Trade Commission (FTC) announced that it was starting to take action against a publisher that was accused of deceiving its authors [24]. There is still great concern that the disappearance of the main available list of PJ, used by several international organizations around the globe, will lead to a larger increase in the number of articles published in these journals. Fortunately, awareness about predatory publishing and journals has been growing. Average proportions of general web queries about PJ have increased between 2014 and 2017 (Figure 1). According to publication trends in Scopus and PubMed databases, formal awareness has also grown with explicit concerns about open access publications (Figure 2).

## Recommendations for researchers

Authors, particularly researchers with little or no previous experience with international publications, need to be careful about the selection of possible journals for submission of their manuscripts due to the current existence of large numbers of PJ. This is particularly important for authors from low- and middle-income countries where we see rapid progress in the global reach of health sciences and the striking limitation to accessing and publishing academic papers in these regions [25].

While it is important to publish results of research – as well as an ethical duty to do so – authors should be aware of issues concerning publication ethics. Since it is likely that the number of PJ will continue to increase (Figures 1 and 2), individual researchers need to be aware of this issue, and carefully assess the quality of potential journals before submitting their manuscripts [26].

Verification of indexing in well-known and high-quality databases, such as Medline, Scopus, and Journal Citation Reports, is an important step. There is an international initiative called “Think. Check. Submit.” (http://thinkchecksubmit.org) that provides several recommendations to potential authors. Authors should look at the journal website and review some of the articles published in the journals to assess their quality; this quick review may be all that is needed to identify PJ. Authors should be aware of the large negative effects, on their careers and on global science, of publishing articles in journals that lack an adequate quality of peer review [27]. Researchers should be strategic in their review of potential journals to avoid the downsides of PJ and select the most appropriate journal for submission of their manuscript.

In addition, researchers need to consider the possible effects of accepting invitations to be external reviewers or members of the editorial boards of PJ. Being asked to serve on an editorial board or as an editor of a journal is a recognition of one’s expertise; however, before accepting any invitation, it is critical to assess the quality of the journal, as serving in the editorial board of PJ is useless as well as detrimental to the researcher’s career.

More research is needed about the factors that influence researchers from developing and developed countries to submit their manuscripts to PJ and the motivations that underlie scientists serving as reviewers or editors of these journals. Additional research is needed about the key factors in scientific quality that differentiate PJ from other journals, in order to develop novel strategies that control the growth and negative impact of PJ on global health research.

## Suggestions for scientific organizations

There are a number of scientific organizations, such as the Committee on Publication Ethics (COPE), the Directory of Open Access Journals (DOAJ), the International Committee of Medical Journal Editors (ICMJE), the Society for Scholarly Publishing (SSP), the Open Access Scholarly Publishers Association (OASPA), and the Association of Learned & Professional Society Publishers (ALPSP), among others, that might generate new approaches to stop the growth of PJ. There is the need for the development of collaborative list that highlight journals that have inappropriate processes for peer review. In addition, multiple indexing services need to identify those journals with questionable editorial processes. As an interesting example from another field, information security researchers have created a web portal entitled “PhishTank” (https://www.phishtank.com/). Individuals can report suspicious cases by using this portal, and experts inspect them and verify real cases of phishing.

Universities around the world, particularly in developing countries [28], might develop strategies to discourage their researchers from submitting manuscripts to PJ or serving as members of their editorial committees. One strategy, adopted by several universities, is that publications in those journals do not count for the academic evaluation or promotion of professors [29]. Organizations that fund research and that evaluate and rate researchers (such as the national science councils) need to highlight the negative consequences of publishing in PJ. Finally, as PJ represent commercial activities based on a possible fraud, national and international entities that are focused on vigilance of financial activities should investigate in more details these companies.

## Conclusions

In conclusion, PJ have shown an exponential growth in recent years, publishing a large number of articles from authors around the globe, with very low quality in the peer review process. Although there have been several initiatives to control or mitigate the negative effects of PJs on health research, it is a growing problem around the world. Unethical publishers create problems for authors particularly inexperienced authors [30]. One interesting solution to avoid the growth of PJ would be the broad existence of much lower fees for researchers from developing countries to publish in recognized OA journals or the availability of more public or private funds to cover these costs. Researchers in global health and other fields need to be aware of PJ and the potential effects of publishing in these journals. Articles about predatory publishing such as this one educate readers to this problem.
